# Non-Native Structures of Apomyoglobin and Apoleghemoglobin in Folding Intermediates Related to the Protein Misfolding

**DOI:** 10.3390/molecules28093970

**Published:** 2023-05-08

**Authors:** Chiaki Nishimura, Takeshi Kikuchi

**Affiliations:** 1 Faculty of Pharmaceutical Sciences, Teikyo Heisei University, Tokyo 164-8530, Japan; 2 Department of Bioinformatics, College of Life Sciences, Ritsumeikan University, Kusatsu 528-8577, Japan; tkikuchi@sk.ritsumei.ac.jp

**Keywords:** apomyoglobin, apoleghemoglobin, protein folding, folding intermediate, quench flow, hydrophobic buried surface area

## Abstract

Protein folding is essential for a polypeptide chain to acquire its proper structure and function. Globins are a superfamily of ubiquitous heme-binding α-helical proteins whose function is principally to regulate oxygen homoeostasis. In this review, we explore the hierarchical helical formation in the globin proteins apomyoglobin and leghemoglobin, and we discuss the existence of non-native and misfolded structures occurring during the course of folding to its native state. This review summarizes the research aimed at characterizing and comparing the equilibrium and kinetic intermediates, as well as delineating the complete folding pathway at a molecular level, in order to answer the following questions: “What is the mechanism of misfolding via a folding intermediate? Does the non-native structure stabilize the contemporary intermediate structure? Does the non-native structure induce slower folding?” The role of the non-native structures in the folding intermediate related to misfolding is also discussed.

## 1. Introduction

Sperm whale apomyoglobin, containing 153 amino acids and with an isoelectric point of 8.7, is arranged in 8 helices (A–H) which, together, form the classic globin fold. Since the protein has no native cysteine residues or disulfides, it has been widely used as a typical model protein for protein folding studies. The F-helix of apomyoglobin remains highly flexible, even in its native state [1,2]. The changes in the structural conformation of the helices have been investigated in circular dichroism studies [3,4]. In addition, the two tryptophan residues Trp 7 and Trp 14 (located in the A-helix) have been used as convenient probes for fluorescence studies. In addition, folding and unfolding studies in combination with molecular biology studies have revealed that kinetic and equilibrium intermediates [5,6,7] are composed of the helices A, G, and H.

Apomyoglobin has been the focus of protein folding studies because of its relatively slow folding process, on a time scale of micromilliseconds. There are several advantages of observing the early stages of protein folding; the starting point is more difficult to observe in detail. Under weakly acidic conditions (pH 4), the apomyoglobin structure forms an intermediate at equilibrium, which can be characterized more easily than a kinetic intermediate. However, the disadvantage is the unavailability of the 3D structure of heme-free apomyoglobin.

The secondary structural elements in protein structures showing differences between α-helical and β-strand structures are important for understanding protein folding. For instance, the α-helices are stabilized by the hydrogen bonds formed between the carbonyl and amide moieties every 3.6 residues; this characteristic aids in speeding up the conformational search process for the native structure. In contrast, the conformation search process for β-structures is time-consuming and complicated because of the long-range interaction involved in the formation of the β-sheet structure. This is related to the backbone topology [8].

Comparison between kinetic and equilibrium apomyoglobin is important for the study of protein folding. An equilibrium intermediate is gained by the adjustment to a pH of 4. Therefore, pH pulse labeling is, essentially, a substitute for a kinetic experiment, whereas the conventional HD experiment is employed for equilibrium. Both systems show amide proton protection against solvents. Therefore, HD exchange patterns are compatible. The only a small difference is observed, especially at the B-helix region. Therefore, similar data were expected between the two studies. Both kinetic and equilibrium structures may be observed as “Molten-Globule” in apomyoglobin.

Non-native structural folding intermediates are observed during the protein folding process. They differ from native-like structures in their secondary and tertiary structural arrangements. Occasionally, a non-native structure can lead to misfolding; the existence of non-native structures in the intermediate forms remains unclear. However, a possible explanation is that non-native structures contribute to the formation of more stable intermediates. Alternatively, they may also induce faster folding or be related to misfolding. Amyloid and aggregate structures are misfolded forms derived from these folding intermediates. Myoglobin can form amyloid fibrils [9]. In this review, we focus on the non-native structures of folding intermediates. It was surprising to find that apomyoglobin (mutant) forms amyloid. Under special conditions (high temperature and pH), the relationship between non-native structures and amyloid formation is still not clear from the mutant study, so it is supposed that further mutant studies will be beneficial. New methodologies are being widely developed, including AFM, in-cell NMR, tweezer, and microinjection for protein science. However, regarding apomyoglobin folding, the information derived from such experiments is limited, especially from studies on the folding intermediate [10] and AFM myoglobin [11].

## 2. Experimental Methods Used in Folding Studies

Stopped-flow instruments are useful for understanding real-time protein folding. For protein folding studies, small volumes of denatured protein solution and a dilution buffer, filled in separate syringes, are mixed rapidly and continuously in a stopped-flow system. After mixing, the reaction was monitored using the (Trp) fluorescence and circular dichroism. Hydrogen–deuterium (HD) exchange can also be used in combination with NMR [12] and mass spectrometry [13]. The HD exchange of amide protons occurs when the amino acid residues are exposed to the solution. To collect snapshot structures initiated by the protein folding reaction, pH-pulse-labeling HD exchange has been used in the study of the kinetics of the folding process. DMSO has been used to stop the reaction after pH-pulse-labeled HD exchange [6]. For protein folding studies, a continuous flow mixer has also been developed [14]. The volumes between the onset of folding and the labeling points are related to the dead time required to chase the reaction. The dead time for the new mixer was 400 μs, compared to 6 ms for the commercially available quench–flow instrument [15].

## 3. Apomyoglobin and Apoleghemoglobin

**Figure 1:**
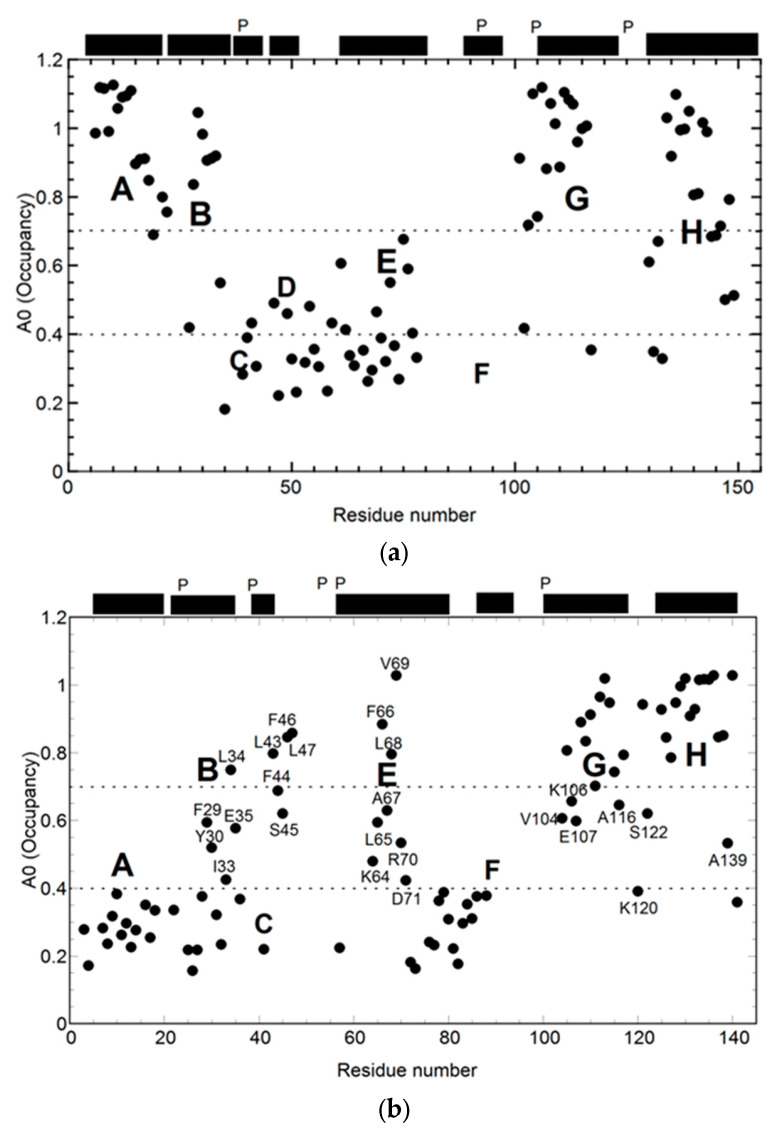
Proton occupancy of the burst phase intermediates (6 ms) of apomyoglobin (**a**) and apoleghemoglobin in which the transition of the first phase trace can be unobservable (**b**): pH-pulse-label quench–flow experiments were conducted, and the HD exchange was monitored using NMR. A0 is the proton occupancy calculated from several experimental data fittings. The data indicate that the intermediates are composed of helices ABGH and EGH for apomyoglobin (**a**) and apoleghemoglobin (**b**) (some residue numbers are included), respectively. The black bars represent the predicted helix locations. “p” indicates the location of Proline. These figures were reproduced with modifications based on the original literature [7,15].

Kinetic and equilibrium folding intermediates of apomyoglobin were identified by pH-pulse-labeling HD exchange experiments at helices ABGH and AGH, respectively [5,6] (Figure 1a). In particular, the B helix is considered a weak point that acts as a trigger between the native and intermediate states [16].

Soybean apoleghemoglobin is similar to apomyoglobin in its native structure and function. Therefore, in the folding pathway of apoleghemoglobin in soybean plants, the intermediate structure is expected to be similar to that of apomyoglobin. However, the former comprises helices E, G, and H instead of helices A, B, G, and H (Figure 1b) [17]. The E-helix plays an important role in the bridging helices G and H. The helix units E to H may be a common subunit in the whole protein’s 3D structure space [18,19] Although the folding topologies of both proteins are similar in their native states, the structures of the intermediates are different.

**Figure 2:**
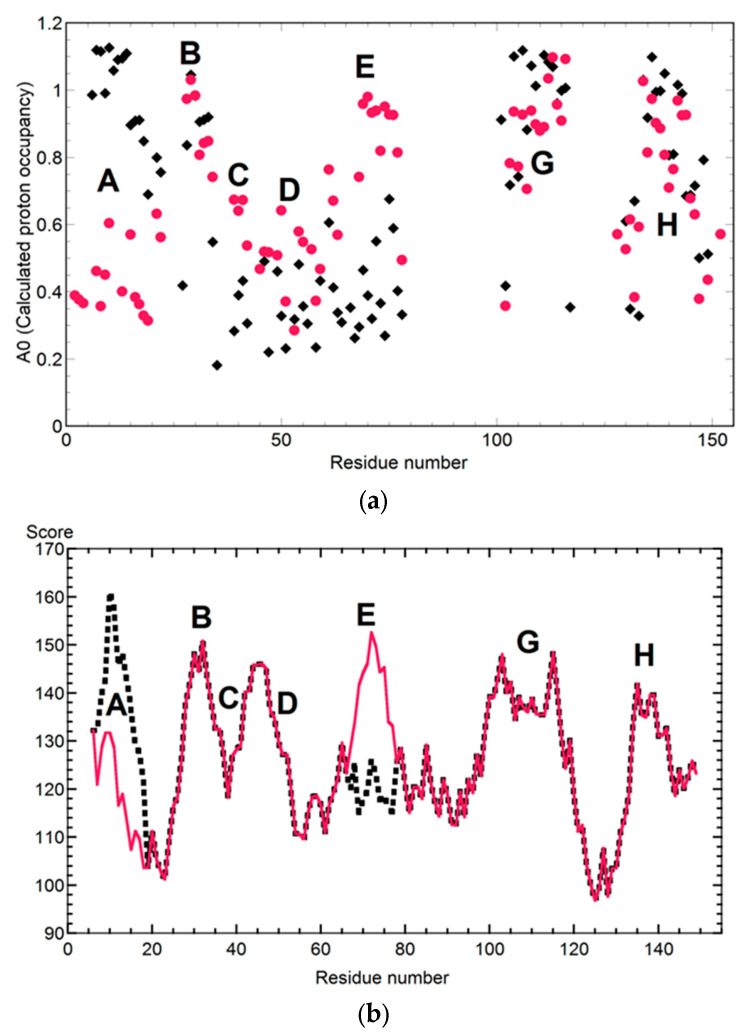
Folding switches to form apoleghemoglobin-like apomyoglobin by mutations of L11G/W14G/A71L/G73W (red) and WT (black). The destabilized A-helix and stabilized E-helix were designed by an average area buried upon folding (AABUF). The AABUF was used for the prediction of the intermediate structure. It was calculated on the basis of the crystal structures. (**a**) Quench–flow experimental data at the burst phase intermediate for L11G/W14G/A71L/G73W (red filled circle) and WT (black filled circle). (**b**) AABUF prediction to protect the burst phase intermediate for L11G/W14G/A71L/G73W (red) and WT (black dash). These plots were revised with some modifications based on the original literature [20].

Apoleghemoglobin-like apomyoglobin intermediate was produced by L11G/W14G/A71L/G73W mutations (Figure 2a) [20]. Switch mutations of the A-helix (L11G, W14G) and E-helix (A71L, G73W) were established by altering the hydrophobic, bulky residues. As predicted by the change in the amino acid sequence, the mutation stabilized the E-helix and destabilized the A-helix. The average area buried upon folding (AABUF) [21] (Figure 2b) was used to predict the intermediate structures [20]. In this system, good agreement was observed between the AABUF prediction and experimental quench–flow data.

**Figure 3:**
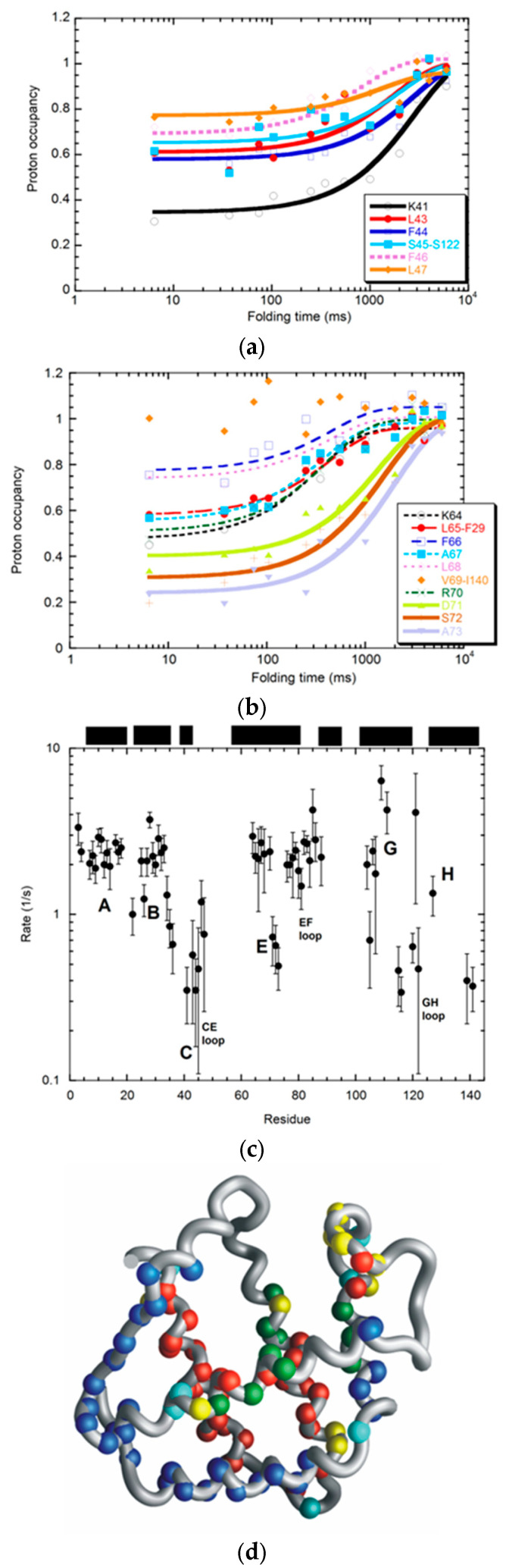
Quench–flow experiments on apoleghemoglobin, monitored by HD exchange followed by NMR detection: transition of proton occupancy of residues in the C-helix and CD-loop (**a**) and E-helix (**b**), wherein two phases can be observed in the regions (thick lines (slow) and dashed lines (fast)). (**c**) Rate constants from the intermediate to the native state: The black top bars correspond to the locations of helices. Error bars are included. (**d**) The folding kinetics of apoleghemoglobin are indicated on the crystal structure of holo-leghemoglobin (1bin.pdb). Red sphere, exchange protected; blue sphere, less protected; and yellow sphere, slower folding (second phase). These plots and mappings on the structure were reproduced, with some modifications from the original literature [7]. Molmol was used for the description of the structure [26].

In addition, two kinetic phases were observed during the folding process of apoleghemoglobin (Figure 3) [7], whereas only one burst phase was observed in apomyoglobin. It is unclear whether parallel or sequential pathways, such as the slower phase, are involved in the folding of apoleghemoglobin. It has been reported that a hidden intermediate may exist in the folding pathway of apomyoglobin [22,23]. Due to the fact that it folds more slowly than that of apomyoglobin, a non-native structure may exist in the intermediate of the apoleghemoglobin pathway. The sequential pathway was proven for apomyoglobin folding based on a double jump experiment [24,25].

## 4. Core and Heme Binding Regions in Apomyoglobin Mutants

The hydrophobic side chains (Leu, Val, and Ile) were substituted with Ala to study their effect on the dynamics of the hydrophobic core formation in the intermediate. Based on the data on the effects of mutation, the intermediate structure was supposed to be slightly altered compared to the native structure. The most interesting observation is that the H-helix was shifted by one helix turn [27,28]. This may have a significant relationship with the misfolding of apomyoglobin.

The F-helix is not folded even in the native state, or has chemical exchange at the native state which induces a missing signal at the F-helix region [1,2]. F2 (P88K/S92K) and F4 (P88K/A90L/S92K/A94L) are stabilizing mutants of the F-helix, which corresponds to the heme-binding region [1,29]. Although these mutations stabilize the protein, its folding kinetics are slower than that of the wild type (WT). This is probably because the folding intermediate is stabilized to a greater extent than in the native structure.

## 5. Rate of Apomyoglobin-Folding

Mutant studies are the most powerful tools for studying protein folding mechanisms. The folding kinetics of many mutants have been determined using the stopped-flow technique. The rate constants were calculated for comparison with those of the WT (3.4 s^−1^), and folding kinetics were categorized as either slow or fast (Table 1). Hydrophobic and bulky side chains were selected in the helices ABEGH, since in the intermediate state, the bulky side chains are suitable because they can form hydrophobic interactions. B- and F-helix mutants were designed for stabilization; these can be the key helices for the intermediate and native states, respectively. It is likely that slower folding can be non-native in nature, whereas faster folding is more similar to native structures. For phi value analysis, folding and unfolding were examined as functions of denaturant concentration using the stopped-flow technique [30]. The structures of the intermediates and transition states during protein folding were examined using Phi-value analysis.

Slower folding rates were observed for V10A (A-helix), I28A (B-helix), L69A (E-helix), L72A (E-helix), F2 (P88K/S92K), and F4 (P88K/A90L/S92K/A94L) (F-helix). Faster folding rates were observed in G23A/G25A, H24L/H119F, I30A, L32A (B-helix), and H64F (E-helix) [31]. It is likely that the sensitive residues in the folding mutations are located in the B and E helices. This is consistent with the fact that the intermediate is composed of ABGH helices. Furthermore, when the mutation causes non-native folding that is connected to misfolding, the folding kinetics may slow down.

## 6. Fluorescence Studies

**Figure 4:**
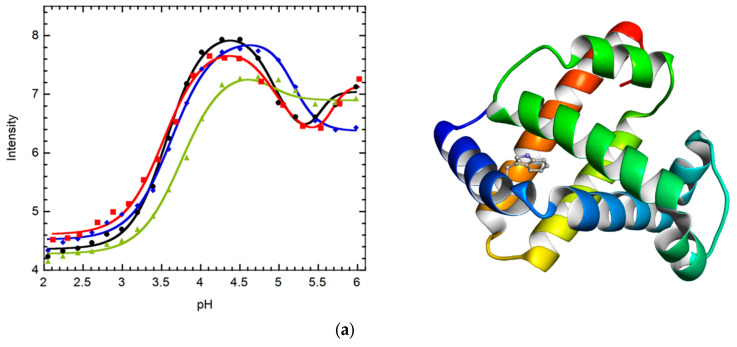

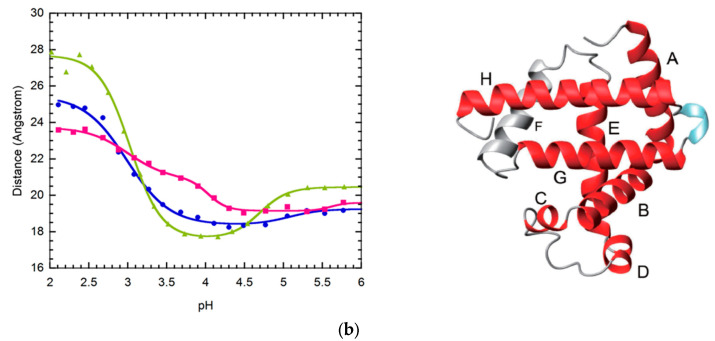
(**a**) Cys-quench of Trp14 fluorescence as a function of pH: Trp7 was mutated by Phe (black). The other mutations, W7F/M131C (blue), W7F/L135C (red), and W7F/I111C (green), were performed to monitor the W14 emission quench induced by Cys. (**b**) Fluorescence resonance energy transfer from W14 to IAEDANS (blue 102, red 105, and green 140) was incorporated by Cys. These figures were produced with modifications based on the original literature [27]. The structure of holomyoglobin is indicated (1mbc).pdb).

The content of the secondary structure was measured by CD, whereas the distances between local regions were measured by FRET, and Trp fluorescence was also used for the newly incorporated Cysteine quench experiments. The size and shape were calculated by Rg with SAXS. Two Trp residues were present in apomyoglobin. Of these, Trp7 was mutated to phenylalanine (W7F) by fluorescence or FRET for evaluation so that the fluorescence quenching or FRET signals could be produced in the proximity of only W14 and the cysteine quenching group or the AEDANS fluorescence acceptor coupled at various sites in the E, G, and H helices. The distance between W14 (A-helix) and the incorporated Cys (G- and H-helices) was measured using cys-quench experiments to monitor the changes in the structure (Figure 4a). The transition of W14 intensity data for I111C and M131C occurred similarly, but that for L135C differed. For W7F/L135C, quenching occurred most efficiently at pH 4, whereas it did not increase at pH 5–6, indicating that the distance between W14 and C135 was in close proximity to the wide pH range of 4–6 [27].

Fluorescence energy transfer (FRET) was examined between W14 and K102C, E105C, and K140C modified by IAEDANS (Figure 4b) [27]. FRET occurred most efficiently in the native state at pH 6, except for with K140-IAEDANS. In this case, FRET occurred most efficiently in the intermediate state (pH 4), indicating that some non-native structures exist between helices H and A. It is likely that the location of the H-helix is not native to the intermediate. Both fluorescence studies clearly indicated that a non-native structure existed in the intermediate.

## 7. Early Stage of Protein Folding

The HD exchange experiment for amide protons was used for sperm whale apomyoglobin at the atomic level [14,32]. A urea-jump experiment was employed for refolding at pH 6, and a quick HD exchange was conducted using continuous-flow experiments. At least, the H-helix was formed as a non-native structure. The H-helix docked helices A and G, and the hydrophobic core was the driving force for helical formation. Non-native structures may be good for the formation of the core. Currently, it is possible that this intermediate structure contains a misfolding structure. At the initial stage of folding, the residues in the A- and G-helices were significantly more protected than those in the other regions.

A new intermediate was identified using the newly developed strategies, especially the continuous mixer: an intermediate L between intermediates I and M. Xu et al. employed a continuous flow device for analyzing whale sperm apomyoglobin folding and unfolding, and conducted urea-jump experiments under weakly acidic conditions. The folding process was monitored by Trp fluorescence and circular dichroism. This experiment and curve fitting showed that two other intermediates (I and L) were observed between U (unfold) and M (intermediate). These intermediates (I, L, and M) were observed at 40 μs, 100 μs, and 250 μs, respectively. Furthermore, sequential pathways were observed for U, I, L, and M. In addition, two intermediates (I and L) were found to be obligatory in this pathway [33].

The other intermediates were observed at the early stage of protein folding (submilliseconds) with the new mixer. The discovery of new intermediates is dependent on the dead time in the improved mixer. Due to the development of a new mixer, new intermediates were identified (I and L).

Chemical modification is a powerful tool for labeling the exposed regions of protein molecules. The pH jump was employed for the initiation of horse apomyoglobin folding, and after the duration of folding, oxidizing labeling was conducted. The exposed regions were selectively labeled via oxidation. The oxidized area was calculated using mass spectrometry. The oxidized regions exhibited heavier molecular weights than the areas kept under unoxidized conditions. This method identified the folding information at the submillisecond range. It revealed that helices A and G were folded within 0.2 ms. Partial folding of helices E and H also occurred. At 0.8 ms, helices A, G, H, E, and B formed, and strong interactions could be observed between helices A and G [34].

An experiment using chemical modification with oxidation was also conducted under different pH conditions at equilibrium, including pH values of 2, 4, and 7 [35]. The three pH conditions corresponded to the acid’s unfolded, intermediate, and native states. At the intermediate state of pH 4, it was observed that helix G was the most protected among the eight helices. The helices A, B, and H were protected, but less so than the G-helix. In the native state of pH 7, the F-helix was not completely unfolded.

The combination of mutant and rapid mixing studies has elucidated the structures of the folding intermediates as well as those of the transition states. The folding and unfolding analysis was monitored via urea-jump experiments by changing the concentration of urea indicated in the V-shape feature (phi-value analysis). Alanine scanning mutagenesis was conducted for the whale sperm apomyoglobin, and rapid mixing was used for folding and unfolding studies. The transition of the structural changes was monitored using Trp-fluorescence and CD with continuous flow. For phi-value analysis, urea-jump experiments were conducted between the unfolded and M states under weakly acidic conditions (U, I, L, and M). Stepwise folding was observed toward the intermediate structure, and helices A, G, and H were gradually stabilized [30].

To examine apomyoglobin folding in real time using the NMR measurement, an azobenzene-modified *cis*-*trans* isomer exchange method was established using peptides [36]. The structures of the isomers were regulated in an NMR probe by direct UV light emission.

U and N correspond to unfolded and native states. Several intermediates were reported, such as Intermediates I, L, and M. In order to observe the intermediates at the early stage of folding, the pH values used provided slightly acidic conditions. Phi-value analysis is a powerful method because it also reveals information about the transition states.

## 8. Computational Analyses on Globin Proteins

In this review, we describe how the NMR H/D exchange technique, stopped-flow technique, FRET, etc., revealed the differences between the folding intermediates apomyoglobin and apoleghemoglobin. The mutants of these proteins on A or E helices exhibit the switch of myoglobin- or leghemoglobin-type folding intermediates. In this section, we briefly discuss the possibilities of computational techniques and whether they can reproduce the phenomenon of folding of globin proteins observed in the aforementioned experiments.

The recent advances in molecular dynamics (MD) simulation techniques are remarkable [37,38], and relatively large proteins have become targets of MD simulations. However, a target of an MD simulation tends to be limited to a relatively small protein for analyses of the folding of proteins [39,40,41]. Concerning globin proteins, the applications of MD techniques are limited to the dynamics of native structures [42,43].

Interestingly, Kolinski et al. [44] already applied a computational technique, namely, coarse-grained Monte Carlo simulation, to apomyoglobin and apoleghemoglobin just after the works published by Jennings and Wright [3] and Nishimura et al. [9] (1993 and 2000). They found that the amplitudes of fluctuations of the E-helix and the beginning of the G-helix dramatically increased as the temperature of the system increased in apomyoglobin. This observation corresponded to the significant fluctuation around the E-helix in the NMR experiments discussed in the present review. They were also able to reproduce the result that the helices A, G, and H were the most stable in the entire apomyoglobin protein, as is consistent with the NMR experiments. The work by Kolinski et al. [37] was conducted in 2003, and the technique used was coarse-grained one, but their results reflected some of the actual behaviors of globin proteins.

Recently, Dasmeh and Kepp [45] performed all-atom MD simulations for holomyoglobins from four mammals. They obtained the result that helices A, G, and H had higher stability, which is consistent with the case of apomyoglobin in the present review.

Another computational field is bioinformatics or amino acid sequential analyses. In the present study, one of the sequence-based analyses, AABUF, was used to predict areas of H/D protection in the sequence of a protein. However, in general, it is rather difficult to extract information on protein folding from its sequence by means of standard bioinformatics techniques.

One of the methods to predict protein folding is based on the statistical information on the 3D structures of proteins. Our technique is based on the statistics of average values of distances between each pair of amino acid types. The studies by Matsuoka et al. [10] can be referenced for the details of this method, but the outline of the method is as follows. An inter-residue average distance of between a specific pair of residue types was calculated using a set of proteins with known 3D structures. In this case, inter-residue means the inter-Cα atomic distance between a pair of residues. Then, a ranking of average distances between pairs of amino acid types was made, taking the separation of two amino acids along the sequence of a protein into account. Then, for a protein with an unknown 3D structure, a plot was made on the map in a way similar to a contact map, where the average distance between two residues is less than a threshold determined in advance. The thresholds or cutoff distances are tuned so that the density of the plots on a map constructed based on inter-residue average distances is close to that of the contact map constructed from the 3D structure of the protein under consideration. A map constructed in this way is called an average distance map (ADM). It should be noted that ADMs are constructed from only the sequence of a protein. A region with a high density of plots along the diagonal of a map can be identified on ADM. Such a region can be regarded as a predicted compact region, which would be structured in the early stage of folding.

The ADMs constructed from the sequences of sperm whale myoglobin and soybean leghemoglobin predicted regions corresponding to the helices A–B and G–H, and helices A–B and E–H as compact regions, respectively. These results denote that the early stages of the folding of these proteins are similar, but some differences appear around the E-helix even if the native structures of both proteins are similar [10,11]. These results correspond well to the results of the present NMR H/D exchange experiments. That is, the folding intermediate structures of these proteins are similar, but in the folding of leghemoglobin, the E-helix is significant in the bridging of the helices G and H.

Thus, as discussed so far, some of computational works can reproduce the experimental features of protein folding at least for globin protein systems. Furthermore, analyses of amino acid sequences also present some features of protein folding. The differences in folding of apomyoglobin and apoleghemoglobin must be remarkable and strongly encoded in their sequences.

## 9. Conclusions

**Figure 5:**
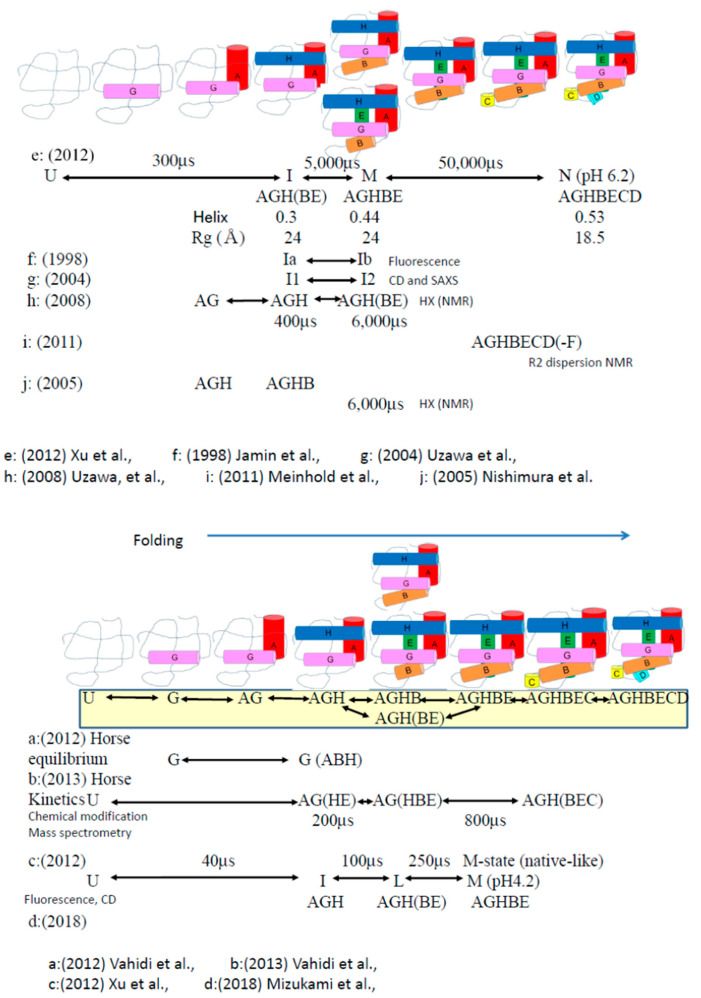
Indicated stepwise folding of apomyoglobin on the basis of the experimental data, including fluorescence, circular dichroism, and chemical modification combined with stopped-flow, pH-pulse-labeling mass spectrometry, NMR, and small-angle X-ray scattering. According to the molar ellipse in CD, helical and beta-strand structures contained in proteins were calculated. From SAXS data, Rg was calculated. Another possible parameter was R_H_. For example, AGH indicated a helical structure composed of helix A, helix G, and helix H. Rate constants for the kinetic study are included. The formation of helices are represented by colors: G (pink), A (red), H (blue), B (orange), E (green), C (yellow), and D (light blue), sequentially [14,20,22,24,30,32,33,34,35].

Apomyoglobin folding is summarized below based on the aforementioned results (Figure 5). The folding reaction is initiated by docking between helices A and G. The presence of a hydrophobic indole ring can contribute significantly to the initial stage of folding. Next, helix H appears to be linked to the stabilized hydrophobic core to form AGH helices. These reactions occur during the early stages of apomyoglobin folding. Furthermore, the B and E helices must participate in helical formation. The mutant for B and E helices decreased the folding speed. These regions may be related to the formation of a non-native intermediate structure. This result is in good agreement that the slow folding was established by a B- and E-helix mutation; it was suggested that both the B- and E-helix mutants significantly delayed the folding process. This is because of the destabilized structure formed in the intermediate. Furthermore, the C- and D-helices docked to a helical structure composed of ABEGH helices. Finally, an F-helix was formed in the presence of heme.

The native structures and functions are similar among the globin proteins. Because the intermediate structures observed were different from the quench–flow HD exchange studies, either of the intermediates at least contains a non-native structure. Based on the folding rate of both proteins, apoleghemoglobin is slower in the last phase. Therefore, it is likely that this occurs via a non-native pathway, which was observed for apoleghemoglobin.

We also revealed a non-native pathway, including H-helix sliding, in apomyoglobin folding based on genetic and fluorescence studies. Protein misfolding may be induced by a non-native structure. Amyloid is produced by slow misfolding, probably from a partial β-structure. The transiently misfolded structure is temporarily produced in a small population and quickly returns to normal major native structures. Studies on non-native structures have provided indications of protein misfolding.

## Figures and Tables

**Table 1:** Folding kinetics of apomyoglobin [31].

Mutants	Location	Rate
V10A	A-helix	slow
I28A	B-helix	slow
L29A	B-helix	medium
L69A	E-helix	slow
L72A	E-helix	slow
L115A	G-helix	medium
P88K/S92K	F-helix	slow
P88K/A90L/S92K/A94L	F-helix	slow
I30A	B-helix	fast
L32A	B-helix	fast
H64F	E-helix	fast
G23A/G25A	B-helix	fast
H24L/H119F	B-helix	fast

## Data Availability

Not applicable.

## References

[1] Nishimura C., Dyson H.J., Wright P.E. (2011). Consequences of stabilizing the natively disordered f helix for the folding pathway of apomyoglobin. J. Mol. Biol..

[2] Eliezer D., Wright P.E. (1996). Is apomyoglobin a molten globule? Structural characterization by NMR. J. Mol. Biol..

[3] Kay M.S., Baldwin R.L. (1996). Packing interactions in the apomyglobin folding intermediate. Nat. Struct. Biol..

[4] Loh S.N., Kay M.S., Baldwin R.L. (1995). Structure and stability of a second molten globule intermediate in the apomyoglobin folding pathway. Proc. Natl. Acad. Sci. USA.

[5] Hughson F.M., Wright P.E., Baldwin R.L. (1990). Structural characterization of a partly folded apomyoglobin intermediate. Science.

[6] Nishimura C., Dyson H.J., Wright P.E. (2005). Enhanced picture of protein-folding intermediates using organic solvents in H/D exchange and quench-flow experiments. Proc. Natl. Acad. Sci. USA.

[7] Nishimura C., Dyson H.J., Wright P.E. (2008). The kinetic and equilibrium molten globule intermediates of apoleghemoglobin differ in structure. J. Mol. Biol..

[8] Gillespie B., Plaxco K.W. (2004). Using protein folding rates to test protein folding theories. Annu. Rev. Biochem..

[9] Fändrich M., Forge V., Buder K., Kittler M., Dobson C.M., Diekmann S. (2003). Myoglobin forms amyloid fibrils by association of unfolded polypeptide segments. Proc. Natl. Acad. Sci. USA.

[10] Elms P.J., Chodera J.D., Bustamante C., Marqusee S. (2012). The molten globule state is unusually deformable under mechanical force. Proc. Natl. Acad. Sci. USA.

[11] Bakke C.K., Jungbauer L.M., Cavagnero S. (2006). In vitro expression and characterization of native apomyoglobin under low molecular crowding conditions. Protein Expr. Purif..

[12] Jennings P.A., Wright P.E. (1993). Formation of a molten globule intermediate early in the kinetic folding pathway of apomyoglobin. Science.

[13] Tsui V., Garcia C., Cavagnero S., Siuzdak G., Dyson H.J., Wright P.E. (1999). Quench-flow experiments combined with mass spectrometry show apomyoglobin folds through and obligatory intermediate. Protein Sci..

[14] Uzawa T., Nishimura C., Akiyama S., Ishimori K., Takahashi S., Dyson H.J., Wright P.E. (2008). Hierarchical folding mechanism of apomyoglobin revealed by ultra-fast H/D exchange coupled with 2D NMR. Proc. Natl. Acad. Sci. USA.

[15] Nishimura C., Dyson H.J., Wright P.E. (2002). The apomyoglobin folding pathway revisited: Structural heterogeneity in the kinetic burst phase intermediate. J. Mol. Biol..

[16] Nishimura C., Wright P.E., Dyson H.J. (2003). Role of the B helix in early folding events in apomyoglobin: Evidence from site-directed mutagenesis for native-like long range interactions. J. Mol. Biol..

[17] Nishimura C., Prytulla S., Dyson H.J., Wright P.E. (2000). Conservation of folding pathways in evolutionarily distant globin sequences. Nat. Struct. Biol..

[18] Matsuoka M., Fujita A., Kawai Y., Kikuchi T. (2014). Similar structures to the E-to-H helix unit in the globin-like fold are found in other helical folds. Biomolecules.

[19] Kikuchi T. (2022). Decoding an Amino Acid Sequence to Extract Information on Protein Folding. Molecules.

[20] Nishimura C., Lietzow M.A., Dyson H.J., Wright P.E. (2005). Sequence determinants of a protein folding pathway. J. Mol. Biol..

[21] Rose G.D., Geselowitz A.R., Lesser G.J., Lee R.H., Zehfus M.H. (1985). Hydrophobicity of amino acid residues in globular proteins. Science.

[22] Meinhold D.W., Wright P.E. (2011). Measurement of protein unfolding/refolding kinetics and structural characterization of hidden intermediates by NMR relaxation dispersion. Proc. Natl. Acad. Sci. USA.

[23] Meinhold D.W., Felitsky D.J., Dyson H.J., Wright P.E. (2022). Transient On- and Off-Pathway Protein Folding Intermediate States Characterized with NMR Relaxation Dispersion. J. Phys. Chem. B.

[24] Jamin M., Baldwin R.L. (1998). Two forms of the pH 4 folding intermediate of apomyoglobin. J. Mol. Biol..

[25] Jamin M., Yeh S.R., Rousseau D.L., Baldwin R.L. (1999). Submillisecond unfolding kinetics of apomyoglobin and its pH 4 intermediate. J. Mol. Biol..

[26] Koradi R., Billeter M., Wuthrich K. (1996). MOLMOL: A program for display and analysis of macromolecular structures. J. Mol. Graph..

[27] Aoto P.C., Nishimura C., Dyson H.J., Wright P.E. (2014). Probing the non-native H helix translocation in apomyoglobin folding intermediates. Biochemistry.

[28] Nishimura C., Dyson H.J., Wright P.E. (2006). Identification of native and non-native structure in kinetic folding intermediates of apomyoglobin. J. Mol. Biol..

[29] Goodman J.S., Chao S.H., Pogorelov T.V., Gruebele M. (2014). Filling up the heme pocket stabilizes apomyoglobin and speeds up its folding. J. Phys. Chem. B.

[30] Mizukami T., Xu M., Fazlieva R., Bychkova V.E., Roder H. (2018). Complex Folding Landscape of Apomyoglobin at Acidic pH Revealed by Ultrafast Kinetic Analysis of Core Mutants. J. Phys. Chem. B.

[31] Nishimura C. (2017). Folding of apomyoglobin: Analysis of transient intermediate structure during refolding using quick hydrogen deuterium exchange and NMR. Proc. Jpn. Acad. Ser. B Phys. Biol. Sci..

[32] Uzawa T., Akiyama S., Kimura T., Takahashi S., Ishimori K., Morishima I., Fujisawa T. (2004). Collapse and search dynamics of apomyoglobin folding revealed by submillisecond observations of alpha-helical content and compactness. Proc. Natl. Acad. Sci. USA.

[33] Xu M., Beresneva O., Rosario R., Roder H. (2012). Microsecond folding dynamics of apomyoglobin at acidic pH. J. Phys. Chem. B.

[34] Vahidi S., Stocks B.B., Liaghati-Mobarhan Y., Konermann L. (2013). Submillisecond protein folding events monitored by rapid mixing and mass spectrometry-based oxidative labeling. Anal. Chem..

[35] Vahidi S., Stocks B.B., Liaghati-Mobarhan Y., Konermann L. (2012). Mapping pH-induced protein structural changes under equilibrium conditions by pulsed oxidative labeling and mass spectrometry. Anal. Chem..

[36] Nagashima T., Ueda K., Nishimura C., Yamazaki T. (2015). Structure-Correlation NMR Spectroscopy for Macromolecules Using Repeated Bidirectional Photoisomerization of Azobenzene. Anal. Chem..

[37] Lane T.J., Shukla D., Beauchamp K.A., Pande V.S. (2013). To milliseconds and beyond: Challenges in the simulation of protein folding. Curr. Opin. Struct. Biol..

[38] Gershenson A., Gosavi S., Faccioli P., Wintrode P.L. (2020). Successes and challenges in simulating the folding of large proteins. J. Biol. Chem..

[39] Chong S.-H., Ham S. (2023). Evolutionary conservation of amino acids contributing to the protein folding transition state. J. Comput. Chem..

[40] Demakis C., Childers M.C., Daggett V. (2021). Conserved patterns and interactions in the unfolding transition state across SH3 domain structural homologues. Protein Sci..

[41] Yang C., Jang S., Pak Y. (2021). Computational Probing of Temperature-Dependent Unfolding of a Small Globular Protein: From Cold to Heat Denaturation. J. Chem. Theory Comput..

[42] Aharoni R., Tobi D. (2019). Dynamical comparison between Drosha and Dicer reveals functional motion similarities and dissimilarities. PLoS ONE.

[43] Maguid S., Fernandez-Alberti S., Ferrelli L., Echave J. (2005). Exploring the common dynamics of homologous proteins. Application to the globin family. Biophys. J..

[44] Kolinski A., Klein P., Romiszowski P., Skolnick J. (2003). Unfolding of globular proteins: Monte Carlo dynamics of a realistic reduced model. Biophys J..

[45] Dasmeh P., Kepp K.P. (2013). Unfolding simulations of holomyoglobin from four mammals: Identification of intermediates and β-sheet formation from partially unfolded states. PLoS ONE.

